# Increased protein propionylation contributes to mitochondrial dysfunction in liver cells and fibroblasts, but not in myotubes

**DOI:** 10.1002/jimd.12296

**Published:** 2020-08-17

**Authors:** Bart Lagerwaard, Olga Pougovkina, Anna F. Bekebrede, Heleen te Brinke, Ronald J.A. Wanders, Arie G. Nieuwenhuizen, Jaap Keijer, Vincent C. J. de Boer

**Affiliations:** ^1^ Human and Animal Physiology, Wageningen University and Research Wageningen Netherlands; ^2^ TI Food and Nutrition Wageningen Netherlands; ^3^ Laboratory Genetic Metabolic Diseases, Department of Clinical Chemistry Academic Medical Center, University of Amsterdam Amsterdam Netherlands; ^4^ Department of Pediatrics Emma Children's Hospital, Academic Medical Center, University of Amsterdam Amsterdam Netherlands

**Keywords:** mitochondria, oxidative metabolism, post‐translational protein modifications, propionic acidemia, propionylation

## Abstract

Post‐translational protein modifications derived from metabolic intermediates, such as acyl‐CoAs, have been shown to regulate mitochondrial function. Patients with a genetic defect in the propionyl‐CoA carboxylase (PCC) gene clinically present symptoms related to mitochondrial disorders and are characterised by decreased mitochondrial respiration. Since propionyl‐CoA accumulates in PCC deficient patients and protein propionylation can be driven by the level of propionyl‐CoA, we hypothesised that protein propionylation could play a role in the pathology of the disease. Indeed, we identified increased protein propionylation due to pathologic propionyl‐CoA accumulation in patient‐derived fibroblasts and this was accompanied by defective mitochondrial respiration, as was shown by a decrease in complex I‐driven respiration. To mimic pathological protein propionylation levels, we exposed cultured fibroblasts, Fao liver cells and C2C12 muscle myotubes to propionate levels that are typically found in these patients. This induced a global increase in protein propionylation and histone protein propionylation and was also accompanied by a decrease in mitochondrial respiration in liver and fibroblasts. However, in C2C12 myotubes propionate exposure did not decrease mitochondrial respiration, possibly due to differences in propionyl‐CoA metabolism as compared to the liver. Therefore, protein propionylation could contribute to the pathology in these patients, especially in the liver, and could therefore be an interesting target to pursue in the treatment of this metabolic disease.

## Graphical abstract

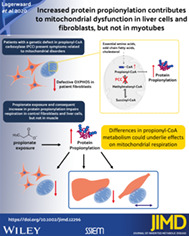

**SYNOPSIS**

Aberrant propionylation contributes to mitochondrial defects and could partly explain the pathology of patients with an inborn genetic defect in the propionyl‐CoA carboxylase gene.

## 1 INTRODUCTION

Post‐translational protein modifications (PTMs) are an important regulatory mechanism for protein functionality and localisation. Modification of proteins offers the cell a rapid and reversible mechanism to respond to changes in the environment, such as changes in metabolite availability. Protein acylation involves the covalent binding of acyl‐groups to lysine residues of a protein and directly links metabolism and protein functionality.^1^ For example, intermediates of metabolism, such as acetyl‐CoA, drive protein acetylation^2^ and can serve as a regulatory mechanism in fatty acid oxidation by acetylation of enzymes involved in the breakdown of fatty acids.^3^

Acetylation is only one type of protein acylation and other acyl‐lysine PTMs have been identified, such as succinylation,^4^ glutarylation,^5^ malonylation,^6^ crotonylation,^7^ butyrylation and propionylation.^8^ Propionylation is the covalent binding of a propionyl‐group to lysine residues of proteins and although being structurally fairly similar to acetylation, the propionyl group is slightly larger and may well be functionally different. Moreover, propionyl‐CoA, the substrate for propionylation, is of special interest, since it is a breakdown product of cholesterol, odd‐chain fatty acids and the amino acids isoleucine, valine, threonine and methionine.^9^ Additionally, propionate is produced by the microbiota and taken up in the intestine.^10^ Propionylation was first identified on histones, where it was later characterised as a transcriptionally active PTM in an in vitro system.^8^, ^11^ Besides histones, propionylation also occurs on non‐histone proteins^12^ and increased propionyl‐CoA levels were able to propionylate the propionyl‐CoA synthetase enzyme in prokaryotes, thereby inactivating it.^13^ This suggests that propionylation of proteins could have a role in metabolic regulation, for example during catabolism and fasting.

Inborn errors in propionyl‐CoA metabolism, such as caused by bi‐allelic mutations in one of the two propionyl‐CoA carboxylase genes (PCC), leads to an accumulation of propionyl‐CoA, a condition known as propionic acidemia (PA). PCC catalyses the carboxylation of propionyl‐CoA into methylmalonyl‐CoA. Methylmalonyl‐CoA can be converted into succinyl‐CoA, which can be used for anaplerosis of the TCA‐cycle.^14^, ^15^ Clinically, the severe neonatal‐onset form of this disease often presents within the first days or weeks in life with encephalopathy, metabolic acidosis and hyperammonaemia and, when left untreated, progresses to coma or death. When treated, the prognosis remains rather poor, with patients showing developmental delay, neurological complications, liver abnormalities, myopathic features and cardiomyopathy.^16^, ^17^ Therefore, more knowledge is required to amend current treatment strategies to improve the disease outcome.

Muscle and liver biopsies from PCC patients show defective mitochondrial respiration, suggesting that mitochondrial dysfunction contributes to the pathology.^18^, ^19^ Interestingly, it is not known how increased levels of propionyl‐CoA can contribute to this. Fibroblasts derived from patients display increased protein propionylation, showing that pathological build‐up of intermediates of propionyl‐CoA metabolism can alter the protein acylome.^20^ We hypothesised that the increased propionylation disrupts normal mitochondrial function and contributes to the mitochondrial phenotype. Here, we use control and patient‐derived fibroblasts and cultured cells to study the mitochondrial effects of increased propionylation to explore a possible role of this PTM in health and disease.

## 2 MATERIALS AND METHODS

### 2.1 Cell culture

Human dermal fibroblasts and Fao hepatoma cells were cultured in Dulbecco's Modified Eagle medium (DMEM) supplemented with 10% (vol/vol) fetal calf serum (FCS), 2 mM glutamine and 1% (vol/vol/vol) pen/strep/fungizone. C2C12 myoblasts were cultured in DMEM supplemented with 10% (vol/vol) FCS, 2 mM glutamine, 25 mM HEPES pH 7.2 and 1% (vol/vol/vol) pen/strep/amphotericin B. Differentiation was induced by replacing medium with DMEM supplemented with 2% (vol/vol) horse serum (HS) upon confluency. Medium was replaced every other day for 5 to 7 days. Propionyl‐CoA carboxylase deficient fibroblasts were obtained from the Gaslini Biobank and C2C12 and Fao cells were obtained from ATCC. Propionate exposure was induced by culturing cells in growth medium containing 4 mM propionic acid from a 400 mM pH‐balanced stock solution. Medium pH remained within the normal range for culture medium (pH 7.3‐7.5).

### 2.2 SDS‐PAGE and Western blotting

Cells were harvested and lysed in TRIS‐HCL pH 7.4 with 1% triton X‐100 containing protease inhibitors and deacylase inhibitors (1 μM trichostatin A and 20 mM nicotinamide). Lysates were sonicated 5 times 2 seconds at 40 kHz amplitude on ice. Protein concentrations were determined using Pierce BCA protein assay kit (Thermofisher) and equal protein amounts were loaded on NuPAGE 4% to 12% gels (Invitrogen), transferred to nitrocellulose membrane, blocked in 3% BSA in PBS with 0.1% Tween‐20 at room temperature and incubated overnight with antibodies in the same buffer at 4°C. Primary antibodies used: β‐actin (#A5441, Sigma‐Aldrich), propionyllysine (#201, PTM biolabs), succinyllysine (#401, PTM biolabs), acetyllysine (#9441, Cell Signalling), histone 3 propionyllysine 23 (#613987, Active Motif), histone 3 acetyllysine 23 (#07‐355, Millipore). IR‐dye based secondary antibodies (LICOR) were used to detect antibody signals using Odyssey scanner (LICOR).

### 2.3 Seahorse mitochondrial respiratory flux analysis

Seahorse XFe96 analysis was performed according to manufacturer's instructions. On the day prior to the analysis, cells were plated at 10 000 cells per well for fibroblasts, 30 000 for Fao and 10 000 for C2C12. For myotubes, 20 000 myoblasts were seeded per well and differentiated in the seahorse plate for 7 days prior to exposure. Before the assay, medium was replaced by XF base medium (Agilent) supplemented with 25 mM glucose and 2 mM glutamine. For analysis of mitochondrial respiration in permeabilised cells, medium was replaced with MAS buffer (220 mM mannitol, 70 mM sucrose, 10 mM KH_2_PO_4_, 5 mM MgCl_2_, 2 mM HEPES pH 7.2, 1 mM EGTA and 0.6% BSA‐fatty acid free) shortly before the assay. Cells were permeabilised by injection of digitonin with concentration of 30 μg/mL for Fao, 37.5 μg/mL for C2C12 and fibroblasts. Oxygen consumption rates (OCR) were analysed following a single injection of pyruvate/malate/ADP (complex I), succinate/rotenone/ADP (complex II), TMPD, ascorbate/ADP (complex IV). Data were normalised to protein concentration or as ratio of nuclei area over background area using 4′,6‐diamidino‐2‐phenylindole (DAPI) staining. For staining: Cells were fixed in 4% formaldehyde for 15 minutes, washed with PBS and stained with 1 μg/mL DAPI for 5 minutes. The BD Pathway 855 microscope (Becton Dickinson; Franklin Lakes, New Jersey) at 10× magnification and an exposure of 0.02. Calculation DAPI pixels over total area in the Seahorse well was done using Adobe Photoshop (Adobe Systems; San Jose, California).

### 2.4 Propionyl‐CoA carboxylase activity

Pellets were resuspended in PBS and sonicated. A volume of 10 μL of protein lysate (1 mg/mL) was added to 40 μL of the reaction mixture (100 mM TRIS‐HCl pH 8.0, 200 mM KHCO_3_, 10 mM MgCl_2_, 10 mM ATP, 1 mM propionyl‐CoA) to a final protein concentration of 0.2 mg/mL. After 15‐minute incubation at 37°C the reaction was terminated with 10 μL of 2 M HCl. The sample was neutralised with 2 M KOH/0.6 M MES buffer after which 30 μL of methanol HPLC grade was added. After centrifugation at 20 000*g* for 5 minutes at 4°C the supernatant was injected on reversed phase HPLC to analyse the formation of methylmalonyl‐CoA.

### 2.5 Propionylcarnitine measurement

Cells were harvested by trypsinization and pellets were resuspended in 0.5 mL demineralised water to 1 mg of protein homogenate. Internal standard (50 PMol ^2^H_3_‐propionylcarnitine) was added to the homogenate, followed by 500 μL of acetonitrile. The samples were vortex‐mixed and centrifuged at 14 000 rpm 4°C for 10 minutes. The supernatant was transferred to a glass vial and the solvent was evaporated at 42°C under a stream of nitrogen. A 100 μL volume of propylation reagent, a 4:1 (vol/vol) mixture of propan‐2‐ol and acetylchloride, was added to the residue, vortex‐mixed and incubated for 10 minutes at 65°C. The propylation reagent was evaporated at 42°C under a stream of nitrogen and the residue was taken up in 100 μL of acetonitrile. Propionylcarnitine was quantified by Electrospray Ionization Tandem Mass Spectrometry (ESI‐MS/MS) as described previously.^21^

### 2.6 Data analysis and statistical testing

Data are presented as mean ± SD. Statistical analyses were performed using GraphPad Prism v.5 (GraphPad Software, La Jolla, California). Means between groups were compared using a Students unpaired *t* test. Significance was accepted at *P* < .05.

## 3 RESULTS

### 3.1 Defective mitochondrial respiration in propionyl‐CoA carboxylase deficient cells

**FIGURE 1:**
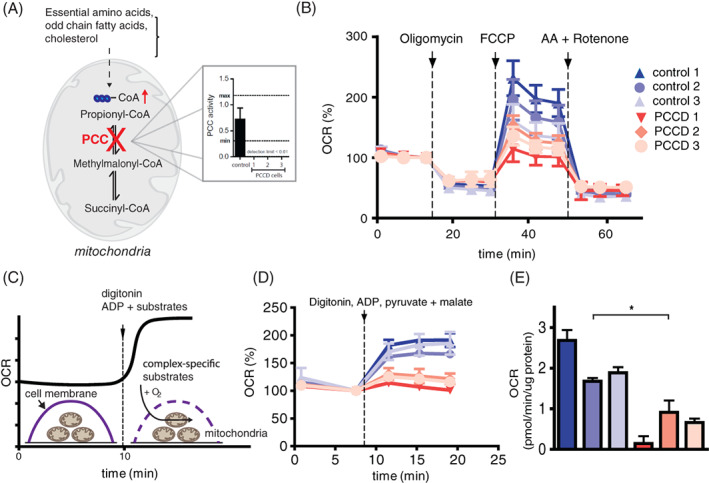
Propionyl‐CoA carboxylase deficient cells have defective mitochondrial respiration. A, Schematic representation of propionyl‐CoA metabolism and its accumulation in propionyl‐CoA carboxylase (PCC) deficient cells and PCC activity in PCC deficient cells. B, Mitochondrial respiration in PCC deficient and control fibroblasts using consecutive injections of oligomycin, FCCP and Rotenone with Antimycin A (AA). C, Schematic representation of the mitochondrial respiration analysis in permeabilised cells. Digitonin was injected together with ADP and complex specific substrates. D, OCR in permeabilised PCC deficient and control fibroblasts with complex I specific substrates, pyruvate and malate. E, Bar graph of complex I linked respiration in PCC deficient and control fibroblasts in the (mean ± SD, * indicates *P* < .05)

To evaluate propionyl‐CoA accumulation and its functional consequences, we used three independent control cell lines and three independent PCC deficient fibroblast cell lines, in which PCC activity was reduced to undetectable levels (Figure 1A). All three patient cell lines had a lower mitochondrial spare capacity compared to controls (Figure 1B). To determine if the decreased OCR was also observed when individual mitochondrial complexes were analysed, we measured respiration in digitonin‐permeabilised cells. The use of an optimised amount of digitonin allows permeabilization of the cell membrane, yet leaving the mitochondrial membrane intact (Figure 1C). We measured OCR following injection of digitonin, ADP and the substrates pyruvate and malate, that are linked to the generation of NADH and complex I respiration. Complex I linked respiration was lower in all the three patient cell lines compared to three control cell lines expressed as increase in OCR after injection of digitonin, ADP and complex I linked substrates (Figure 1D). When comparing normalised maximal complex I linked respiration, there was a significant difference between control and patient cell lines, demonstrating the defective mitochondrial respiration in these PCC deficient cells (Figure 1E).

### 3.2 Exposure to propionate provokes a mitochondrial defect in control fibroblasts

**FIGURE 2:**
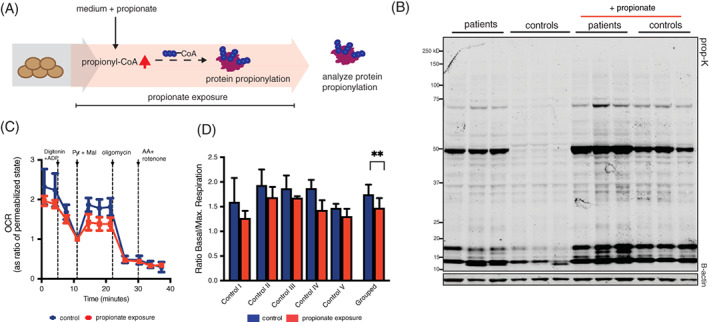
Inducing propionylation imitates mitochondrial respiratory defects observed in PCC deficient cells. A, Schematic representation of the experimental set‐up. B, Anti‐propionyllysine western blot analysis of three control and patient fibroblasts in control medium and exposed to 4 mM propionate for 3 weeks. C, Representative measurement of mitochondrial respiration of control cell line exposed to propionate or control medium for 10 days. Medium was replaced with control medium day before analysis. Cells were permeabilised with digitonin and ADP, pyruvate and malate were added to induce complex I‐driven respiration. Normalised OCR data is expressed as a ratio from basal respiration, determined as OCR after digitonin injection. D, Ratio basal to maximal complex I respiration for five control cell lines exposed to control or propionate medium (mean ± SD, ** indicates *P* < .01)

During PA, propionate accumulates in plasma to concentrations as high as 5.4 mmol/L, a thousand fold higher than in healthy individuals.^22^ To mimic this in vitro, we exposed control cells to long‐term extracellular propionate. Control and patient fibroblasts were cultured in medium containing 4 mM of propionate for 3 weeks (Figure 2A). We previously showed that a state of metabolic PA significantly induces protein propionylation.^20^ Indeed, Protein propionylation was increased in patient as compared to control cells and after exposure to propionate, propionylation in both the patient and control cells was increased significantly (Figure 2B). Furthermore, profiling of mitochondrial respiration showed that exposure to propionate significantly reduced complex I‐driven respiration in five fibroblast control cell lines (Figure 2C,D). This suggests that long‐term propionate exposure could contribute to mitochondrial pathophysiology in PCC deficient cells, possibly via aberrant protein propionylation.

### 3.3 Exposing Fao cells to propionate induces protein propionylation and causes decreased mitochondrial respiration

**FIGURE 3:**
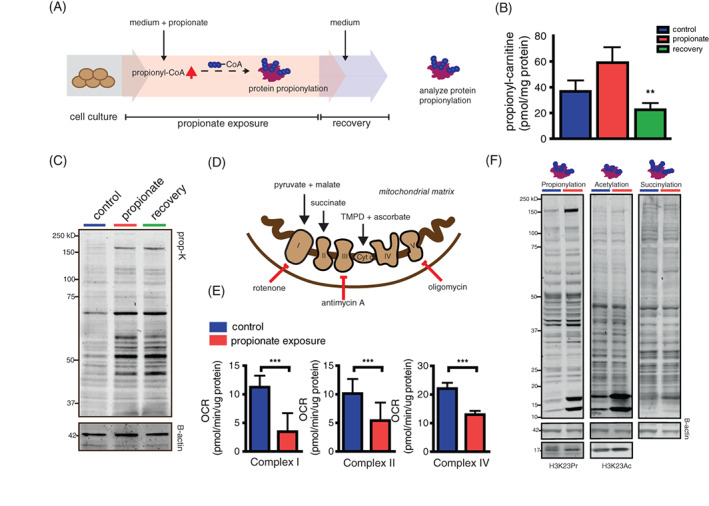
Exposure to propionate induces protein propionylation and decreases activity of mitochondrial respiratory complexes in Fao liver cells. A, Schematic representation of the experimental set‐up. Fao cells were exposed to 4 mM propionate for 5 days. This was followed by 1‐day recovery step to ensure that propionate and propionyl‐CoA levels would decline to normal in the cells. B, Propionylcarnitine levels in the cells after propionate exposure and after the recovery. C, Anti‐propionyllysine western blot analysis of cell lysates cultured in regular medium, after propionate exposure and after recovery. D, Schematic representation of mitochondrial complexes with specific substrates and inhibitors that were used. To measure the activity of respiratory complexes pyruvate and malate (complex I), succinate and rotenone (complex II), TMPD + ascorbate (complex IV) were injected together with digitonin and ADP in permeabilised control cells and cells exposed to propionate. E, Quantification of complex respiration control cells and cells exposed to propionate. F, Antipropionyl, −acetyl, −succinyl, histone 3 lysine 23 propionyl (H3K23Pr) and histone 3 lysine 23 acetyl (H3K23Ac) western blot analysis of cell lysates exposed to control or propionate medium for 7 days (mean ± SD, ** indicates *P* < .01, *** indicates *P* < .001)

The liver is exposed to propionate that is produced and taken up from the colon. Levels in the portal vein can reach concentrations between 17 and 194 μmol/L.^10^ To test whether we can induce protein propionylation in the liver cells, we exposed Fao rat hepatoma liver cells to 4 mM propionate for 5 days. We included 1 day of recovery in regular medium after the exposure, before harvesting the cells (Figure 3A), to ensure that propionyl‐CoA and propionate levels in the cells were not elevated, and the effects of increased protein propionylation rather than increased propionate and/or propionyl‐CoA levels would be analysed. Because propionylcarnitine profiles accurately reflect cellular propionyl‐CoA levels^23^ and our propionyl‐CoA analysis was not sensitive enough to determine propionyl‐CoA levels in cultured cells, we monitored propionylcarnitine levels. After 5‐day propionate exposure, propionylcarnitine levels were increased. Notably, after 1 day of recovery, propionylcarnitine levels decreased and normalised to the same levels as in the cells that were not exposed to propionate (Figure 3B). This implies that 1 day of culturing on regular medium is sufficient to remove excess cellular propionate. Interestingly, 1 day recovery on propionate‐free medium did not lower protein propionylation levels compared to cells exposed for 5 days without a recovery day (Figure 3C). Thus, our experimental set‐up yields cells with increased protein propionylation and baseline propionyl‐CoA levels and eliminates any confounding effects of propionate, or its derived metabolites, on inhibition of mitochondrial enzymes. Functionally, propionate exposure and increased propionylation was accompanied by a decrease in mitochondrial respiration in permeabilised Fao cells looking at complex I, complex II and complex IV driven respiration (Figure 3D,E).

Liver perfused with 5 mM propionate show significant changes in other acyl‐CoA species, such as succinyl‐CoA.^24^ Therefore, we tested whether exposure to propionate increases other protein acylations, such as acetylation or succinylation. We observed an increase in succinylation and acetylation in response to propionate exposure, yet this increase was limited to histone proteins (Figure 3F), Additionally, we measured acetylation and propionylation of histone 3 lysine 23 (H3K23) to verify if indeed these changes altered acylation on these specific sites. Propionylation and acetylation of H3K23 was increased after exposure to propionate (Figure 3F). This shows that exposure to propionate can alter other acylations in the cell, although these acylations are not similar in every cell compartment.

### 3.4 C2C12 myotubes exposed to propionate show increased propionylation, but do not display defective mitochondrial respiration

**FIGURE 4:**
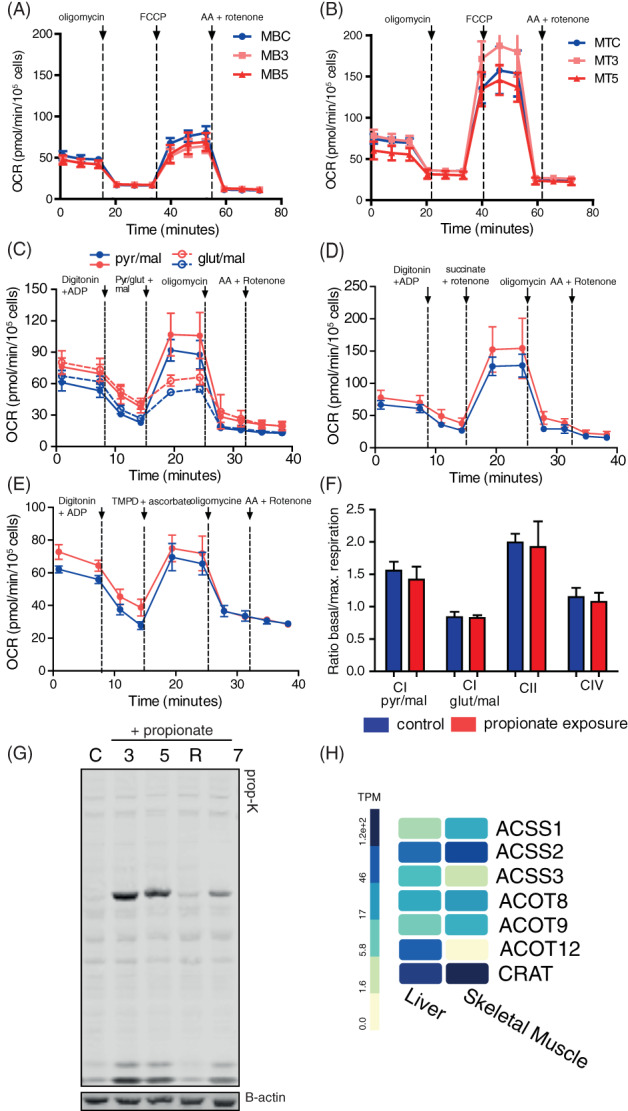
C2C12 muscle cells exposed to propionate show increased propionylation, but do not display defective mitochondrial respiration. Mitochondrial respiration in myoblasts, A, and myotubes, B, in control (MBC/MTC), after 3 days (MB3/MT3) and after 5 days of propionate exposure. Respiration was measured using consecutive injections of oligomycin, FCCP and Rotenone with Antimycin A (AA). Respiration of individual complexes was measured in permeabilised myoblasts after 5 day of propionate exposure and overnight exposure to control medium day before assay for complex I, C; complex II, D; and complex IV, E. F, Quantification of complex respiration control cells and cells exposed to propionate (mean ± SD). G, Anti‐propionyllysine western blot analysis of cell lysates cultured in regular medium and after 3‐, 5‐ and 7‐day propionate exposure and after 1 day of recovery in regular medium. H, Expression of selected genes in propionyl‐CoA metabolism for skeletal muscle and liver. Expression data obtained from Genotype‐Tissue Expression (GTEx) Portal.^26^ Gene and transcript expression are shown in Transcripts Per Million (TPM). Acyl‐CoA synthetase short chain family member 1 (ASSC1), acyl‐CoA synthetase short chain family member 2 (ASCC2), acyl‐CoA synthetase short chain family member 3 (ASCC3), acyl‐CoA synthetase medium chain family member 1 (ACSM1), acyl‐CoA synthetase medium chain family member 3 (ACSM 3), acyl‐CoA thioesterase 8 (ACOT8), acyl‐CoA thioesterase 9 (ACOT9), acyl‐CoA thioesterase 12 (ACOT12), carnitine acetyltransferase (CRAT)

Apart from neurological, haematological and hepatic complications, patients with PA may also display myopathic features, including hypotonia and exercise intolerance.^16^, ^25^ Therefore, we used a murine C2C12 muscle cell model combined with propionate exposure to test whether protein propionylation could contribute to these features. Interestingly, both C2C12 myoblasts and myotubes exposed for 3 or 5 days to propionate did not show defective mitochondrial respiration (Figure 4A,B). Also, complex I (Figure 4C), Complex II (Figure 4D) and complex IV (Figure 4E) driven respiration were not reduced. Neither, when accounting basal respiration by calculating the ratio between basal over maximal respiration (Figure 4F). Myotubes exposed to propionate did show increased propionylation levels, but to a lesser extent than we observed in Fao cells. Moreover, a 24‐hour recovery period reduced propionylation levels significantly (Figure 4G), indicating that propionate handling in muscle can be distinct from liver and fibroblasts. Indeed, propionate and propionyl‐CoA handling genes were differently expressed between muscle and liver tissue, as was analysed using the Genotype‐Tissue Expression (GTEx) portal (Figure 4H).

## 4 DISCUSSION

The aim of this study was to assess the role of protein propionylation in the aetiology of PA and in cultured cells. We show that fibroblasts of PCC deficient patients displayed increased propionylation of the of proteins throughout the cell, including histones. PCC deficient fibroblasts as well as propionate‐exposed control fibroblasts and Fao cells showed a decreased mitochondrial respiration. Since propionylation could impact protein function directly in the mitochondria or indirectly via histone modification, the observed mitochondrial dysfunction could be attributed to elevated protein propionylation in PCC deficient patient cells and could thus play a role in the pathology of PA. In contrast, propionate exposure in C2C12 myotubes did not affect respiration. C2C12 myotubes showed propionylation, although to a lesser extent. Furthermore, there was faster de‐propionylation after propionate exposure was removed in C2C12 myotubes compared to Fao cells.

The observed differences in the effects of propionate exposure on mitochondrial respiration between our liver and muscle model may be explained by the intrinsic differences in propionate handling. The liver is exposed to higher physiological concentrations of propionate compared to the rest of the body.^10^ While in muscle, propionyl‐CoA is not primarily derived from propionate, but instead comes mainly from the breakdown of amino acids.^15^ This is in line with differences in transcriptional regulation of enzymes involved in acyl‐CoA metabolism in muscle and liver. The only known specific propionyl‐CoA synthetase, acyl‐CoA synthetase short chain family member 3 (ACSS3), was lower expressed in muscle than in liver, suggesting a higher activity to convert propionate into propionyl‐CoA.^27^, ^28^ Although ACSS3 is specific for propionate, at higher concentrations the mitochondrial acetyl‐coenzyme A synthetase 2‐like (ACSS1) also has some affinity for propionate.^29^ On the other hand, the major mitochondrial propionyl‐CoA degrading enzyme, acyl‐CoA thioesterase 9 (ACOT9),^28^, ^30^ is higher expressed in muscle than in liver, whereas the cytosolic acyl‐CoA thioesterase 12 (ACOT12) that primarily hydrolyses acetyl‐CoA, but also has some activity towards propionyl‐CoA,^31^ is expressed lower in muscle compared to in liver. Finally, carnitine acetyltransferase (CRAT) has the highest affinity for propionyl‐CoA^32^ and is higher expressed in muscle than in liver. This could indicate that the muscle is likely more capable of lowering mitochondrial propionyl‐CoA levels by conjugation with carnitine and is possibly geared towards propionyl‐CoA elimination. Indeed, Matsuishi et al^33^ showed that exposure of isolated liver mitochondria to 5 mM propionate increased the levels of propionyl‐CoA, which concomitantly decreased the respiratory control ratio, whereas exposure of muscle mitochondria to propionate did not increase propionyl‐CoA and did not affect respiration.^33^ These differences in propionyl‐CoA handling could explain that C2C12 myotube mitochondrial respiration is not affected by propionate exposure, while liver cell mitochondria are more sensitive to propionate exposure, possibly due increased propionyl‐CoA levels and consequent aberrant propionylation.

De Keyzer et al^19^ showed multiple mitochondrial defects in muscle, liver and heart tissues from PA patients and Schwab et al^18^ observed mitochondrial defects in muscle biopsies of two young PA patients, showing a decreased enzyme activity of all mitochondrial complexes. Since we did not observe propionylation‐related defects in mitochondrial respiration in myotubes in vitro, the mitochondrial defects in PA patients in vivo could be unrelated to propionyl‐CoA accumulation and propionylation in muscle mitochondria. Alternatively, it could be that the mitochondrial defects observed in PA patients could be secondary, due to, for example, hampered muscle innervation, leading to muscular hypotonia and physical inactivity.^16^, ^34^

Additionally, it must be noted that our liver and muscle PA model do not truly reflect the metabolic situation in PCC patients. First, using the current model, propionyl‐CoA does not build up inside the mitochondria specifically, as is the case in PCC patients. Second, exposure to propionate in an intact cell system will result in anaplerosis of the TCA cycle at succinyl‐CoA.^24^, ^35^ Therefore, the consequent increase in downstream metabolites might exert its effects on respiration beyond the increase in protein propionylation. For example, exposure to propionate increases levels of methylcitrate, which can inhibit TCA enzymes such as isocitrate dehydrogenase, possibly affecting respiration measurements.^36^ Yet, after infusion of rat liver with 5 mM propionate, levels of methylcitrate did not reach levels close to the inhibition constant, possibly minimising the inhibitory role of this metabolite on cellular respiration.^24^ Other metabolites that are known to accumulate in PA patients, such as 3‐hydroxypropionic acid, have been shown to directly cause respiratory defects in isolated heart mitochondria.^37^ Therefore, although it is currently unknown if these metabolites also accumulate in the current PA model used in liver and muscle, these metabolites could have directly influenced respiration in our models.

Another effect of exposure to propionate is the depletion of free cellular CoA due to the formation of propionyl‐CoA and methylmalonyl‐CoA, also known as CoA trapping.^24^ Incubation with propionate and carnitine increased free cellular CoA and propionylcarnitine, resulting in less accumulation of propionyl‐CoA in heart^35^ and partially reversed mitochondrial defect in liver.^33^ Less accumulation of propionyl‐CoA increases the cellular CoA pool but at the same time could likewise result in lower propionylation of the proteome. Nevertheless, in order to minimise the direct effects CoA trapping and inhibitory effects of products of propionate metabolism on respiration, we incorporated a washout period in our experimental set‐up. This washout allowed propionylcarnitine levels, as a proxy for propionyl‐CoA, to return to control values, while propionylation remained elevated, presumably allowing for the measurement of respiration with little interferences of these metabolites and CoA trapping.

The PCC enzyme is a mitochondrial enzyme and therefore propionyl‐CoA was expected to primarily effect the mitochondrial proteome. However, we show that propionylation in fibroblasts of PCC patients also occurs outside of the mitochondria, since we see increased propionylation on histone proteins. This is accordance with an in vivo Pcca−/− mouse model, that showed increased histone propionylation.^11^ Moreover, in Fao cells increased histone propionylation, as well as increase in histone acetylation was observed when exposed to extracellular propionate. Histone proteins can be subjected to various acyl‐modifications, hereby distinctly regulating chromatin structure and transcription.^38^ This could possibly contribute to the pathology of PCC patients and to the phenotype observed in our PA models. Yet, increased histone propionylation does not seem to cause respiratory defects per se, as histone propionylation was increased in myotubes in absence of decreased respiration. Nonetheless, the effect of propionylation on histone proteins is not yet fully understood. Acetyltransferases, previously identified to primarily acetylate histones, also show propionylation activity^39^, ^40^ and histone acetylation and propionylation appear to be functionally similar as both marks are associated with transcriptional activation.^11^ However, whether histone propionylation is merely an additional transcriptional activator or if there are physiological and pathological situations, such as in PA, in which histone propionylation may have distinct regulatory roles on gene expression is yet to be explored.

We show that increased global protein propionylation and specific histone protein propionylation in liver might contribute to the respiratory defects observed in PA and therefore therapies that aim to reduce or reverse propionylation could be beneficial in the treatment of PA. Liver complications, including hepatomegaly and hyperechoic liver, belong to the most common complications in PA^16^ and mitochondrial defects are thought to contribute to these pathologies, at least in part. In current treatment strategies, PA patients may benefit from liver transplantation, which may even reverse frequent complications, such as cardiomyopathy.^41^ However, the transplantation is associated with high mortality due to complications.^42^ Other treatment strategies include: carnitine supplementation to decrease intracellular propionyl‐CoA levels,^33^, ^43^ restriction of dietary factors that end up as propionyl‐CoA, such as propiogenic amino acids, and the use of an antibiotics to reduce propionate production by gut bacteria. Despite these strategies, the outcome for PCC patients remains poor with significant clinical impairment, such as delayed mental development and episodes of acute metabolic decompensations.^44^, ^45^, ^46^

One explanation for the poor outcome could be that propionyl‐CoA levels are still elevated or elevate acutely due to the endogenous production of propionyl‐CoA.^9^ Therefore, it is of interest to explore alternative strategies that might alleviate the underlying causes that could contribute to the liver pathology, such as aberrant protein propionylation. However, targeting the enzymatic propionylation by acyltransferases would possibly be an ineffective strategy, as much of the propionylation is likely non‐enzymatic.^20^ Hence, to decrease aberrant propionylation we propose to increase de‐propionylation activity in PA patients. Although it is not clear which enzymes regulate de‐propionylation, possibly sirtuins 1‐3 hold some de‐propionylation activity, at least in vitro.^47^, ^48^ In particular activation of sirtuin 3 (SIRT3) could be an interesting strategy due to its cellular localisation within the mitochondria and multiple metabolic targets, including mitochondrial complexes.^49^ SIRT1 could be of interest as a potential strategy to remove aberrant propionylation on histone proteins, as this enzyme has been shown to de‐propionylate proteins, at least in vitro.^12^ Therefore, efforts are required to identify how proteins are de‐propionylated to alleviate aberrant propionylation and its consequences. These efforts could help to improve the treatment of PCC patients and hereby improve the health outcome and quality of life.

## AUTHOR CONTRIBUTIONS

Bart Lagerwaard, Olga Pougovkina, Anna F. Bekebrede and Heleen te Brinke performed experiments, principal data analysis and reporting of results. Bart Lagerwaard, Olga Pougovkina, Anna F. Bekebrede, Heleen te Brinke, Ronald J. A. Wanders, Arie G. Nieuwenhuizen, Jaap Keijer and Vincent C. J. de Boer involved in planning and conduct of the project, conception and design of research, data analysis and interpretation. All authors edited, revised and approved the final version of the article.

## CONFLICT OF INTEREST

The authors declare no conflicts of interest, financial or otherwise. FrieslandCampina and Danone Research B.V are sponsors of the TIFN program and partly financed the project. They had no role in data collection and analysis, decision to publish, or preparation of the article.

## INFORMED CONSENT

This article does not contain any studies with human or animal subjects performed by any of the authors.
